# Delivery of sFIT-1 engineered MSCs in combination with a continuous low-dose doxorubicin treatment prevents growth of liver cancer

**DOI:** 10.18632/aging.101146

**Published:** 2016-12-28

**Authors:** Jian Niu, Yue Wang, Ji Wang, Bin Liu, Xin Hu

**Affiliations:** ^1^ General Surgery of the Hospital Affiliated Hospital of Xuzhou Medical University, Digestive Disease Research Laboratory of Xuzhou Medical University, Xuzhou, Jiangsu 221002, PR China; ^2^ The University of Texas Graduate School of Biomedical Sciences at Houston, MD Anderson Cancer Center, Houston, TX 77030, USA

**Keywords:** liver cancer, gene therapy, mesenchymal stem cells, vascular endothelial growth factor, soluble Fms-like tyrosine kinase-1 (sFlt1)

## Abstract

One important process in liver cancer growth and progression is angiogenesis. Vascular endothelial growth factor (VEGF) has the significant role in liver cancer angiogenesis. sFlt1 (soluble Fms-like tyrosine kinase-1) is the promising inhibitor of VEGF and can be used as the new method of inhibiting angiogenesis. MSCs (Mesenchymal stem cells) can infiltrate into tumor tissue and function as the efficient transgene delivery mediator. Here, we engineered murine MSCs to express sFlt1 and examined the anti-tumor effect of MSC- sFlt1 in combination with continues low-dose doxorubicin treatment. We found that this combination therapy significantly inhibited liver cancer cells proliferation. Above all, HepG2 xenografts treated with this combination therapy went into remission. It is of note that this inhibition effect was not p53 binding and by increasing caspase8. This study suggests that this combination treatment has novel therapeutic potential for liver cancer because of significantly inhibiting cancer cells growth and anti-angiogenesis in vitro and in vivo.

## INTRODUCTION

Despite aggressive treatment with operation and combination with chemotherapy, the prognosis of liver cancer remains poor. Most liver cancers are of rich blood supply and there is the close correlation between angiogenesis and tumor progression [1–2]. Recently, anti-angiogenesis has been proved to be the hopeful strategy for liver cancer treatment [3–4].

VEGF is the significant angiogenic factor and plays its roles by interacting with its receptors including Flt-1 (Fms-like tyrosine kinase-1) and Flk-1 /KDR (fetal liver kinase-1/kinase insert domain containing receptor). sFlt1 is a spliced form of the Flt1 receptor and it comes from the Flt1 transmembrane domain. sFlt1 binds to VEGF as a part of the full-length receptor and this binding blocks signal transduction. Importantly, Flt1 protein is expressed in liver cancer cells [5–8].

Reports have shown that transferring of sFlt1 into tumor can inhibit tumor angiogenesis and growth [9–10]. MSCs can be engineered to express genes and are known to infiltrate into tumor tissues [11–14]. Therefore, MSCs may provide an avenue for sFlt1 delivery in liver cancer.

Doxorubicin leads to DNA damage and cell apoptosis in a lot of cancer cells. Doxorubicin has also the distinct antitumor effect for liver cancer cells. In this study, we used the treatment of continuous low-dose doxorubicin combination with MSC.sFlt1 to demonstrate the additive inhibition of liver cancer cells growth and anti-angiogenesis in vitro and in vivo.

## RESULTS

### sFlt1 is expressed by MSCs and released into the culture media

**Figure 1:**
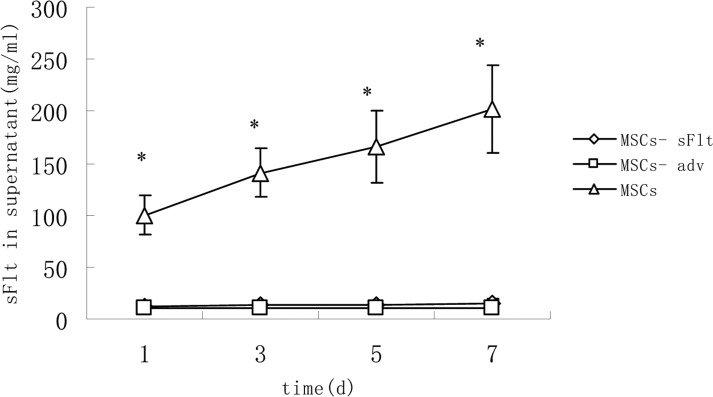
The content of sFlt1 in supernatant increased significantly in MSCs-sFlt1 group as compared with day-matched MSCs-adv group or MSCs group Data from 3 independent experiments are shown. *P<0.05 versus MSC-sFlt1 group.

To measure transient expression of sFlt1 in MSCs, 24 hours after adv-sFlT1 transduction into MSCs, sFlt1 protein (100.0±18.7mg7/ml) was found from cell culture supernatant and the expression of sFlt1 was detected for more than 7 days (Figure 1).

### Endothelial cells are sensitive to low dose doxorubicin treatment

**Figure 2:**
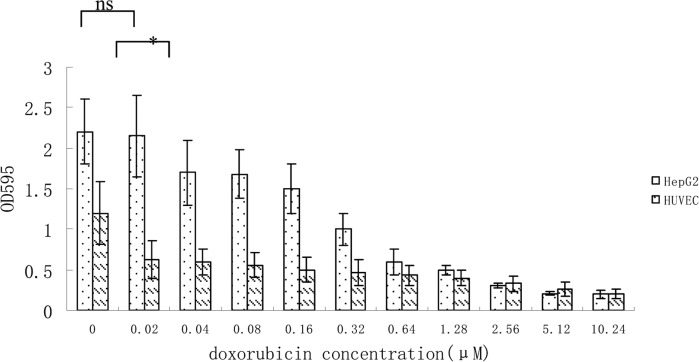
Growth inhibition of HUVEC and HepG2 cells at different concentration of doxorubicin Data from 3 independent experiments are shown. *p < 0.05, n.s. not significant, vs 0.02μM.

Since most endothelial cell assays utilize HUVEC (human umbilical vein endothelial cells) [15], we tested the sensitivity of HUVEC cells to doxorubicin which is widely used in liver cancer chemotherapy. Our data suggested that HUVEC cells proliferation were significantly more inhibited at low-dose doxorubicin (0.02μM) than HepG2 cells (Figure 2).

### Combination treatment had more inhibitory effect on HUVEC cells functions

**Figure 3:**
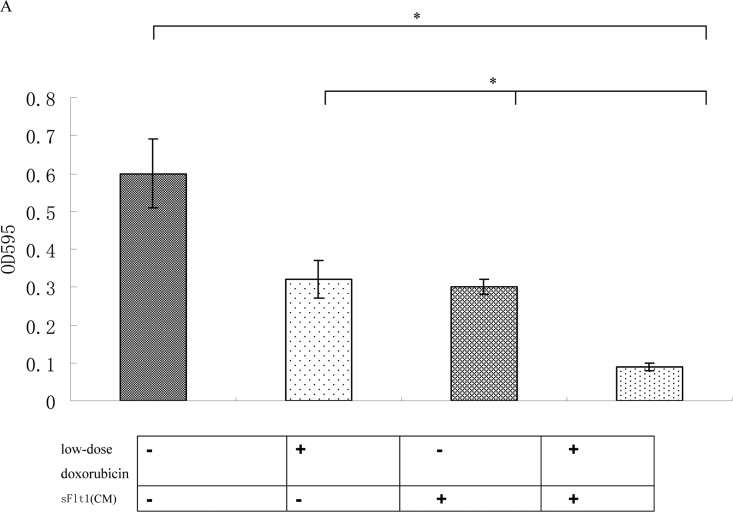

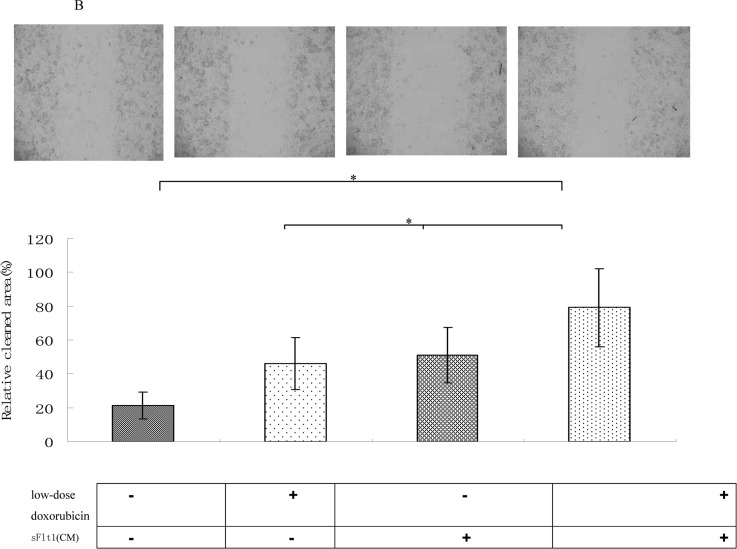
(**A**) The combination therapy enhanced the proliferation inhibition of HUVECs cells in vitro. (**B**) Wound healing assays were performed. The amount of migrating cells of sFlt1 (1.5 ml of concentrated conditioned medium) plus low-dose doxorubicin group were much lower than control. Magnification, 200×. Picture of one representative experiment of 3 is shown. Data from 3 independent experiments are shown. *p < 0.05, vs sFlt1 plus low-dose doxorubicin group.

To prove that sFlt1 engineered MSCs can inhibit the growth of HUVEC cells, we investigated the effect of sFlt1 and/or low-dose doxorubicin on HUVEC cells functions in vitro. The results showed single-agent sFlt1(concentrated conditioned medium) or low concentration doxorubicin inhibited the migration and proliferation of HUVEC cells. Interestingly, the combination therapy exerted a significant enhanced inhibitory effect on HUVEC cells proliferation and migration (Figure 3).

### Combination therapy of doxorubicin and sFlt1 leads to additive apoptosis induction

**Figure 4:**
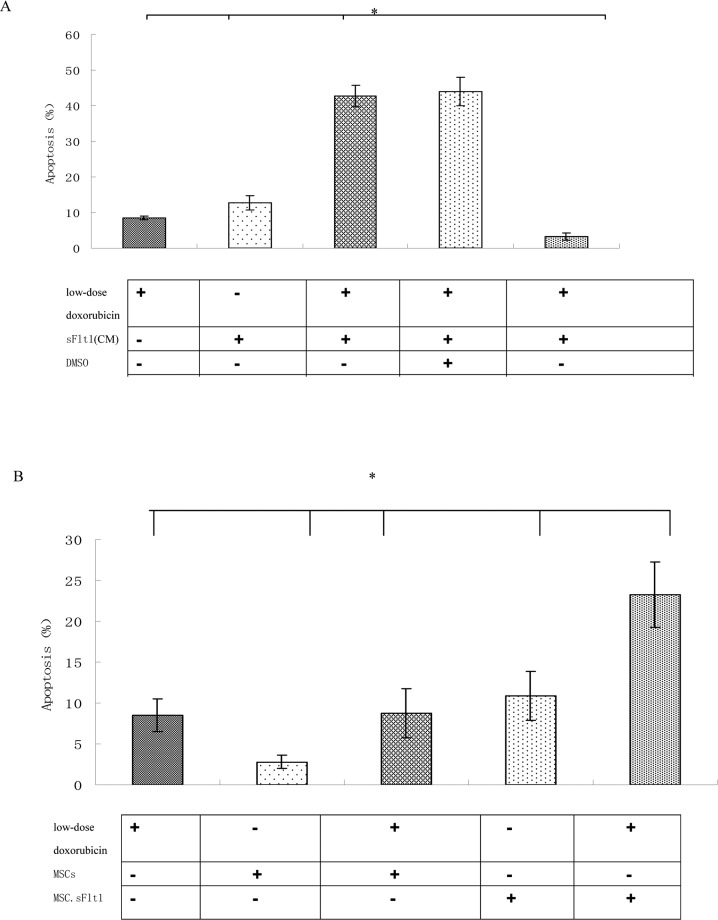
HepG2 cells can be sensitized to low-dose doxorubicin inducing by MSC. sFlt1 (**A**) Apoptosis measurement was conducted after the combination treatment. Addition of zVAD to the HepG2 cells before combination treatment can inhibite apoptosis. (**B**) Mixing HepG2 cells with MSC.sFlt1 (in a ratio of 10:1) plus 0.02μM doxorubicin treated for 48 h showed significantly increased apoptosis as compared with other group. Data from 3 independent experiments are shown. *p < 0.05, vs sFlt1 plus low-dose doxorubicin group.

Because low-dose doxorubicin (0.02μM) alone was not efficient to inhibit HepG2 cells, we began to test sFlt1 -based treatment utilizing combination with low-dose doxorubicin. We treated HepG2 cells with sFlt1 (1.0 ml of concentrated conditioned medium) plus 0.02 μM doxorubicin for 48h before we measured cell apoptosis. This combination therapy resulted in 37±0.6% apoptosis as compared with 2.6±0.4% (sFlt1 group) and 10.5± 0.4% (low-dose doxorubicin group). Apoptosis could be inhibited by zVAD (carbobenzoxy-valyl-alanyl-aspartyl-[O-methyl]-fluoromethylketone), showing that apoptosis was correlate with caspase (Figure 4A).

One important step for clinical use of sFlt1 is effective delivery and sufficient bioavailability in carcinoma. The carcinoma-infiltrating properties of MSCs could be used to deliver sFlt1. We had to determine whether MSCs could effectively deliver sFlt1. To test this in vitro, we mixed MSC.sFlt1 with HepG2 cells for 48 h. This gave rise to apoptosis levels significantly (Figure 4B).

### Combined therapy of MSC.sFlt1 plus continuous low-dose doxorubicin is efficacious and safe *in vivo*

**Figure 5:**
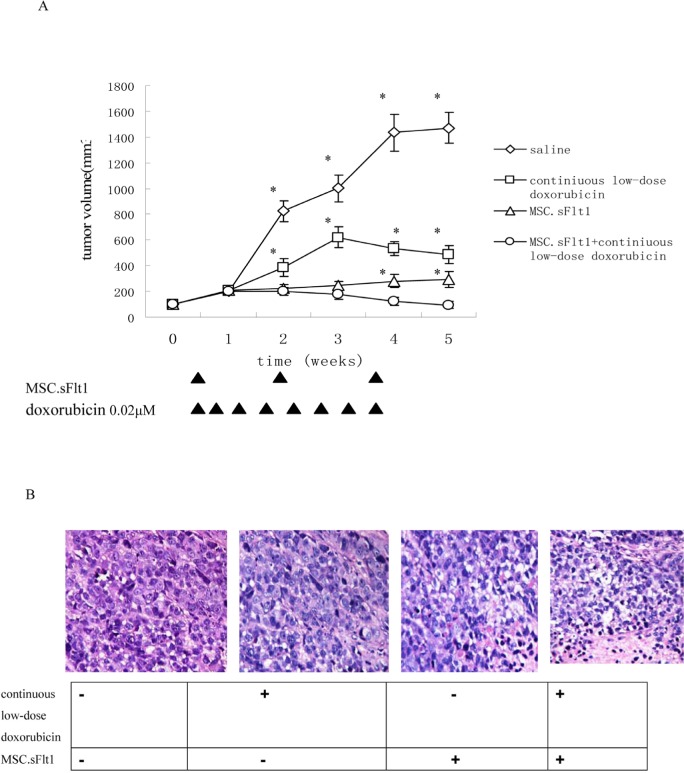

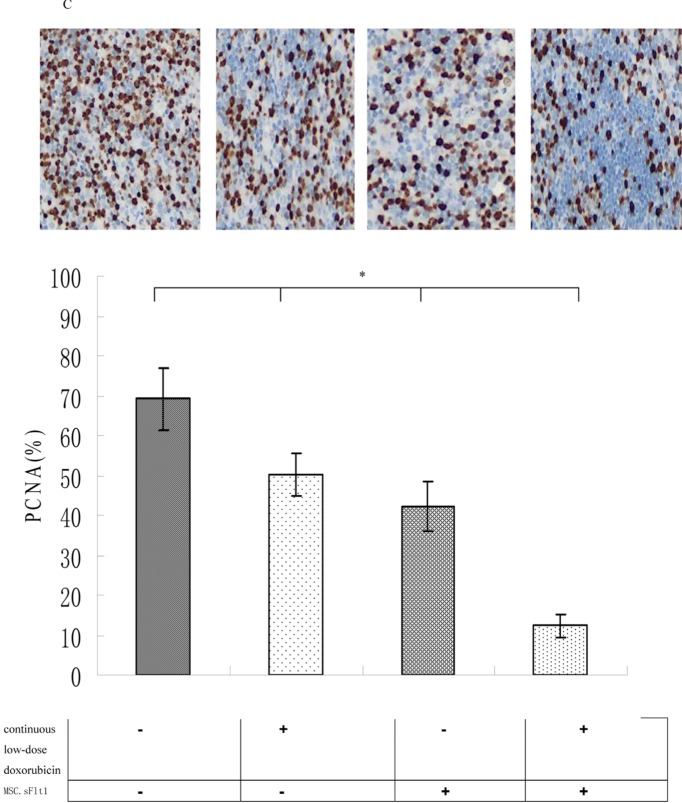

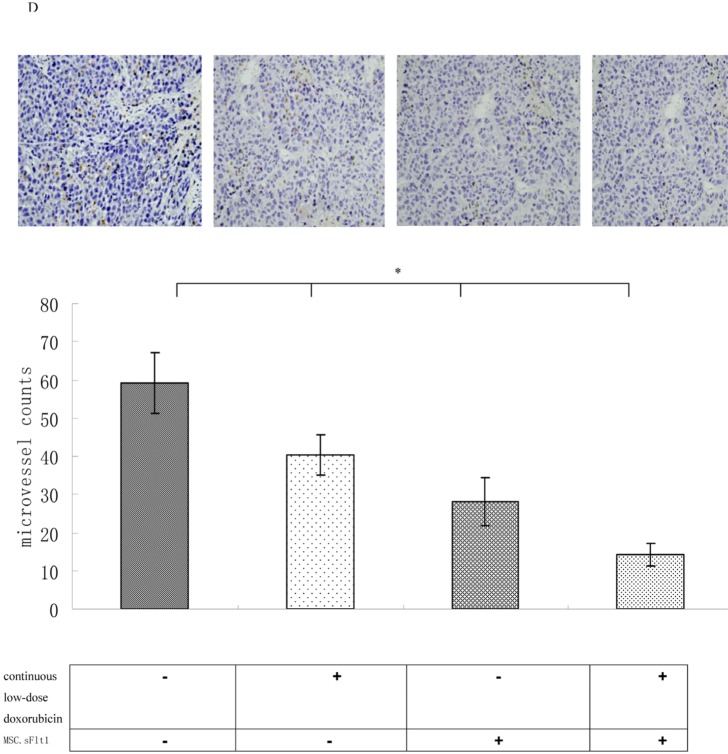

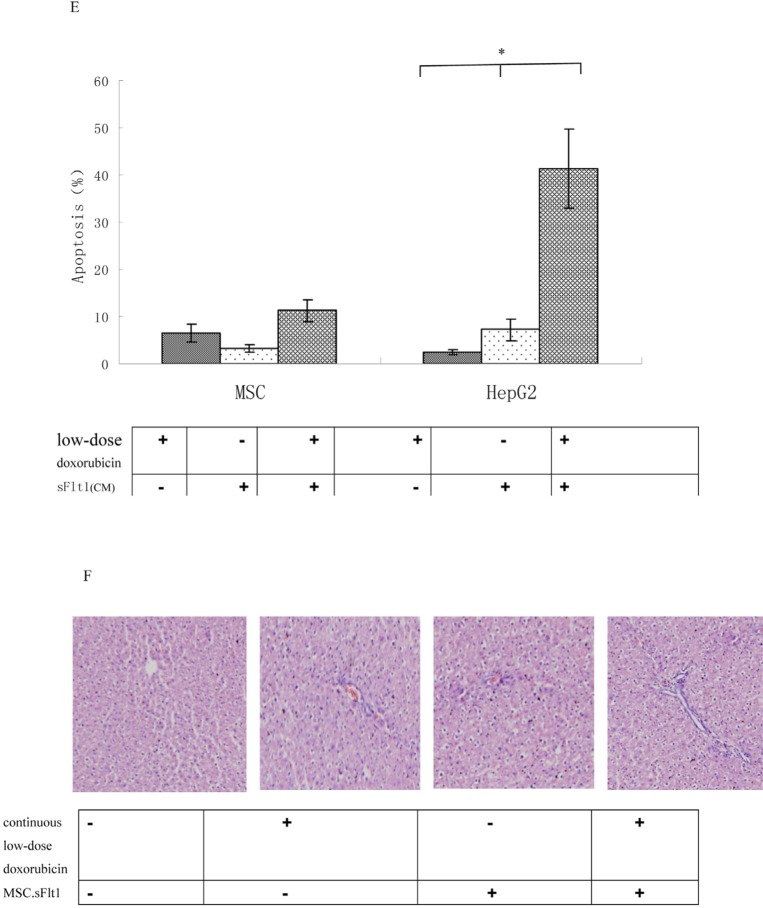
Treatment with continiuous low-dose doxorubicin plus MSC.sFlt1 leaded to tumor remission and was safe in vivo (**A**) Animals bearing HepG2 xenografts began treatments when tumor diameters reached 5 mm as described in “Materials and Methods.” Arrows: days on which treatment was administered. Results are given as mean tumor volume of 7 mice /group. *p < 0.05, vs MSC.sFlt1 plus continuous low-dose doxorubicin group. (**B**) HE staining was to examine general tumor tissue morphology from saline group, continuous low-dose doxorubicin group, MSC.sFlt1 group and MSC.sFlt1 plus continuous low-dose doxorubicin group. Magnification, 200×. (**C**) Immunohistochemical detection of PCNA protein expression (brown color) of saline group, continuous low-dose doxorubicin group, MSC.sFlt1 group and MSC.sFlt1 plus continuous low-dose doxorubicin group. Combination therapy enhanced the inhibition of tumor cell proliferation in vivo. Magnification, 200×. Data from 3 independent experiments are shown. *p < 0.05, vs MSC.sFlt1 plus continuous low-dose doxorubicin group. (**D**) Immunohistochemical analysis for MVD in HepG2 xenografts and combination therapy enhanced the inhibition of tumor angiogenesis in vivo. Magnification, 200×. *p < 0.05, vs MSC.sFlt1 plus continuous low-dose doxorubicin group. (**E**) Apoptosis was measured after exposure of MSCs or HepG2 cells to 0.02μM doxorubicin plus sFlt1 (1.0 ml of concentrated conditioned medium) for 48 h. *p < 0.05, vs sFlt1 plus low-dose doxorubicin group. (**F**) HE staining of liver sections from mice treated with saline, continuous low-dose doxorubicin, MSC.sFlt1 and MSC.sFlt1plus continuous low-dose doxorubicin, respectively. Magnification, 200×.

We established HepG2 xenografts in immune-deficient Balb/c mice. Although the tumors in saline group, grew almost exponentially, xenografts either treated with continuous low-dose doxorubicin or MSC.sFlt1 showed obvious growth inhibition. Surprisingly, xenografts treated with combination therapy went into remission (Figure 5A). Nonencapsulated tumor with cancer cells infiltration was found in sample sections of saline group by HE analysis. In contrast, MSC.sFlt1 plus continuous low-dose doxorubicin group clearly showed cellular necrosis and fibrosis instead of cancer cells (Figure 5B). Moreover, PCNA (nuclear proliferating cell nuclear antigen) protein was detected by immunohisto-chemistry. PCNA levels decreased significantly from the combination therapy group compared with other group (Figure 5C). To determine whether combination therapy inhibit tumor angiogenesis, tissue specimens were immunostained with CD31 mAb to count microvessels density (MVD). The data showed MVD was significantly lower in combination therapy group than other group (Figure 5D).

Then, we detected the safety of combination therapy on MSCs. We incubated MSCs with 0.02μM doxorubicin plus sFlt1 (concentrated conditioned medium) for 48h. MSCs were resistant to the single treatment as well as to the combination therapy by detecting apoptosis (Figure 5E).

Moreover, we detected the liver tissue of mice that had treated with combination therapy and could not detect any signs of tissue damage showing that this treatment is safe relatively (Figure 5F).

### The molecular mechanism of the combination therapy is not p53 binding

**Figure 6:**
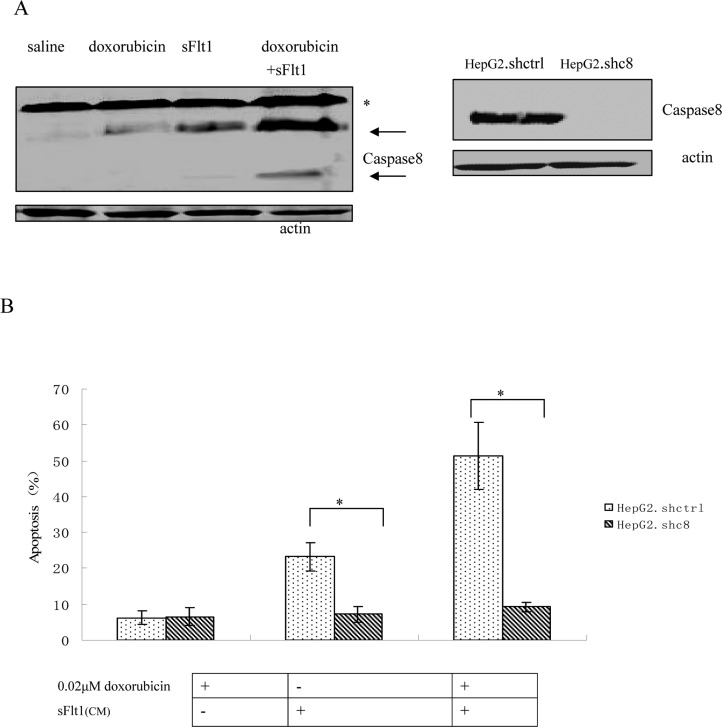

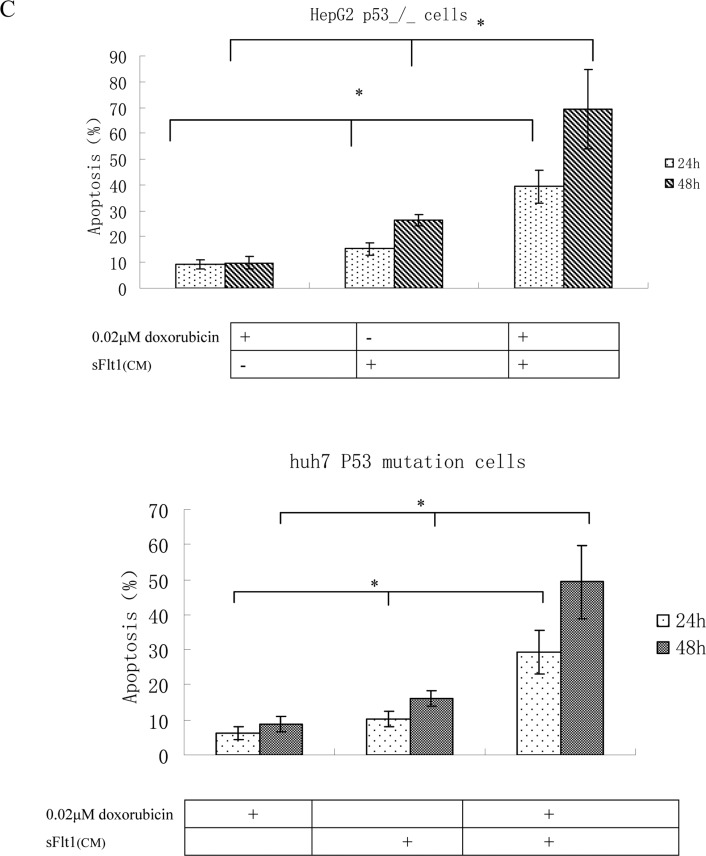
(**A**) Western blot of Caspase8 that HepG2 cells were either treated with saline, 0.02μM doxorubicin, sFlt1, or 0.02μM doxorubicin plus sFlt1 for 48 h. Pro-caspase bands are marked by an (*), whereas Caspase8 are labeled by arrows. (**B**) Caspase8 knockdown clone (HepG2.shc8) and control clone (HepG2.shctrl) were treated with doxorubicin plus sFlt1 before the apoptosis was measured. The apoptosis of HepG2.shc8 was decreased. *p < 0.05, vs sFlt1 plus 0.02μM doxorubicin group. (**C**) Apoptosis measurements in HepG2 p53−/− cells or huh cells after treatment with 0.02 μM doxorubicin plus sFlt1 (concentrated conditioned medium) for 24h and for 48h. HepG2 p53−/− cells and huh7 cells exhibited increased apoptosis in response to combination treatment *p < 0.05, vs sFlt1 plus low-dose doxorubicin group.

To further improve sFlt1-based combination treatments, the better knowing of the molecular mechanisms is necessary. First, caspase8 was tested which is the important molecular event in the apoptosis pathway. Caspase8 activation is markedly increased in sFlt1 treated group compared to saline group. Additionally, caspase8 level increased further in sFlt1 plus low-dose doxorubicin group compared with other groups. In order to further testify that the sFlt1 sensitization effect is by caspase8, we cloned stable cell lines of caspase8 knockdown, named HepG2.shc8 (Figure 6A). HepG2.shc8 were treated with 0.02μM doxorubicin plus sFlt1 for 48 h, then the apoptosis was measured. Results showed that the apoptosis of HepG2.shc8 was decreased, showing that caspase8 is the initiator in this process (Figure 6B). Next, we want to prove the apoptosis induced by the combination treatment is p53 independent. We found that low-dose doxorubicin plus sFlt1-triggered apoptosis reached the same level in HepG2 p53^−/−^ cells as in HepG2 cells. To test that combination treatment-induced apoptosis is not cell specific, we also analyzed huh7 cells, which express p53 mutation, and found that huh7 cells could exhibit increased apoptosis in response to combination treatment (Figure 6C).

## DISCUSSION

As a lot of tumor growth are correlate on angiogenesis, antiangiogenic therapy shows the great promise for tumor treatment.

Recently, scientists found that blocking VEGF/VEGFR signaling pathway can inhibit tumor growth [16, 17]. As we all know, human liver cancer cells produce lots of proangiogenic factors including VEGF/VEGFR that may serve as therapeutic targets. For example, some synthesized drugs in the market by targeting VEGF receptors can suppress the proliferation of liver cancer cells [18–21].

But all of these drugs have many side effects and with only moderate therapeutic activities. The possible explanation is that these drugs have the short half time or limited accessibility to liver cancer tissue.

Tumor-targeted therapy is one of the major strategies for pharmacotherapy of cancer today. sFlt1 is the potent antagonist of VEGF. sFlt1 could selectively incorporate with VEGF and inhibit VEGF activity [22, 23].

As MSCs can infiltrate into tumor tissue, they have been applied in cancer treatment [24–26]. In this study, we successfully constructed sFlt1 engineered MSCs as verified by ELISA assay (Figure 1) and explored the potential of MSC-sFlt to target tumor angiogenesis and growth.

Previous reports showed that withdrawal of neutralizing anti-VEGF antibody treatment resulted in tumor recurrence [16–17]. These experiments proved that antiangiogenic therapy is insufficient and combination with chemotherapy is needed. Antiangiogenic-schedule chemotherapy is recommended because this kind of chemotherapy enable clinicians to give lower doses of drugs and has less side effects [27–28]. This novel strategy is explored as anti-tumor treatment effectively [29–31].

Doxorubicin has the significant anti-tumor effect for human liver cancer, either as a single agent or in combination with other cytotoxic agents [32]. Our data prove that low-dose doxorubicin inhibited HUVEC cells growth significantly more than HepG2 cell growth (Figure 2). And low dose doxorubicin plus sFlt1 can inhibit proliferation and migration of HUVEC cells more efficiently in vitro (Figure 3). Moreover, we observed the direct inhibition effect of this combination treatment on proliferation of HepG2 cells in vitro (Figure 4).

Next, we conducted animal experiments to test whether MSC.sFlt1 plus continuous low dose doxorubicin treatment can induce growth retardation or remission of xenografts with less side effects.

Our data showed that this combination treatment inhibited the growth of HepG2 xenografts in nude mice and had no toxicity as represented by liver histology examination (Figure 5). To prove whether the inhibitory effect was related with anti-angiogenesis, we tested MVD in tumor samples and found this combination treatment led to significant decreasing in MVD compared with other group (Figure 5). The mechanisms of anti-tumor growth of MSC.sFlt1 plus continuous low dose doxorubicin includes the elevated apoptosis of cancer cells and endothelial cells (Figure 4, 5).

HepG2 cells express wild p53 but many kinds of liver cancer cells express p53 mutated and/or dysfunctional. Hence, in order to evaluate the combination treatment, we analyzed huh7 p53 mutated cells and HepG2 p53^−/−^cells. We observed the increased apoptosis following the combination treatment in both cancer cell lines. These results proved that the therapy is effective in cancer cells expressing p53 mutated and/or dysfunctional (Figure 6). Subsequently, we want to clarify the mechanism in this progress to improve the MSC. sFlt1 system. Silencing of caspase8 resulted in the apoptosis inhibition of huh7 p53 mutated cells or HepG2 P53^−/−^ cells. This result showed that the inhibition of cancer cells was correlate with caspase8 (Figure 6).

In conclusion, MSC.sFlt1 plus low-dose doxorubicin therapy effectively suppressed the progression of liver cancer cells with less side effects in the mice xenograft model. The mechanism lies in its anti-angiogenesis and anti-cancer cells effect. This treatment might be more practical for clinical application and can be of the development of the targeted therapy for liver cancer.

## METHODS

### Materials and reagents

Recombinant adenovirus (adv-sFlt) was provided by Dr. S Luo (Zhongsan University, Guangdong, China). Adv-sFlt contained the 1–3 Ig-like domains of Flt according to the sequence information from GenBank (accession No. X51602) [33]. Cell lines HepG2, huh7 and HUVEC were obtained from Chinese national human genome center (Shanghai, China). With regard to P53 status, HepG2 cells carry wild-type P53 and huh7 cells carry mutation P53 [34]. HepG2 cells were maintained in MEM and huh7 cells in DMEM containing 100U/ml of penicillin and 100 μg/ml of streptomycin in a humidified chamber at 37°C in 5% CO_2_. HUVEC cells were cultured in DMEM with 10% fetal bovine serum, 100 unit/ml heparin, and 75 μ*g*/ml of endothelial cell growth supplement (ECGs; SIGMA, Saint Louis, MI) in 5% CO_2_ at 37°C. MSCs from 4 to 6 week old female BALB/c nu/nu mice were prepared based on the method of Peister et al [37]. The cells were grown in Dulbecco's modified Eagle's medium (DMEM)–low glucose with 100 U/ml penicillin, 100ug/ml streptomycin and 10% fetal bovine serum. The mice bone marrow mesenchymal stem cells (BMMSCs) are generally referred to as MSCs throughout the text.

### Generation of secreted MSC-sFlt1 and ELISA

The adenovirus hosting sFlt was introduced into the MSCs at a multiplicity of infection (MOI) of 100 in DMEM for 24 hours with 8μg/mL polybrene. After 24 hours, the collected supernatant was concentrated by a centrifugal filter (Millipore, Schwalbach, Germany) at 4000 rpm for 30 min at 4°C. The concentrated conditioned medium (CM) was then filtered through 0.2 μm filters and stored at −80°C for further use. MSCs were infected with adv-sFlt for 24 hours, then washed twice and the culture supernatant containing secreted sFlt was examined for 7 days. sFlt1 expression was verified via ELISA using a commercial kit (Invitrogen, USA) according to the instructions provided by the manufacturer. The sFlt1 concentration in the cell supernatant samples was calculated based on the standard curve.

### Cell proliferation assay

Cell proliferation were examined by MTT assay [35]. 20 ml of MTT solution (5 mg/ml in PBS) (Aqueous One Solution Assay, Promega, USA) was added to each well, and the cells were incubated for 4 h at 37°C. Then, absorbances were measured at 595 nm using a microtitre plate reader (Titertek Multiskan MCC, USA). The percentage of cell proliferation was calculated by defining the absorption of cells not treated with doxorubicin (control) as 100%. A total of three independent experiments were performed, and the means were used to depict the survival curve.

### Apoptosis assay

Apoptosis was measured as previously described [36]. In brief, 3 × 10^4^ HepG2 cells were cultured in a 24-well plate for 24h. We treated HepG2 cells with sFlt1 (1.5 ml of concentrated conditioned medium, CM) plus 0.02μM doxorubicin for 48h, then we measured cell apoptosis. Cells were collected by trypsinization and washed twice with cold 1× PBS. Then, Nicoletti buffer (Sodium citrate 0.1 % containing 0.1 % Triton X-100) and propidiumiodide (50g/ml) was added to the cell pellets. Tubes were vortexed for 10s at medium speed and left for 5 h in the dark (4°C). The fluorescence intensity was then measured in a flow cytometer (BD, USA).

### Wound healing assay

3 × 10^4^ HUVEC cells were cultured in a 24-well plate for 24 h. After a tight cell monolayer was formed, the cells were incubated with serum-free medium for 24 h and the cell monolayer was wounded with a plastic pipette tip [37]. The remaining cells were washed twice with fresh medium to remove cell debris, and further incubated with 1.5 ml CM for 12 h, purified recombinant human VEGF165 (R&D Systems, Minneapolis, USA) at 10 ng/ml was added to HUVEC cells, which were assayed for migratory ability. After 24h, the migrant cells at the wound front were photographed with a microscope.

### Mixture of MSC-sFlt with liver cancer cells

Tumor cells were plated in six-well plates at 1×10^6^ cells per well and left overnight to adhere. Then, HepG2 cells were treated with 1×10^5^ MSC.sFlt1 and doxorubicin (0.02μM) for 48h (in a ratio of 10:1). Then cells were collected for apoptosis assays.

### Animal studies

The animal study was performed to verify the effects of MSC.sFlt1 plus continuous low-dose doxorubicin in inhibiting the growth of liver tumors in vivo. Balb/c nu/nu mice (4-6 weeks old, 18-20 g body weight) were purchased from the experimental animal center of Shanghai, Chinese Academy of Sciences (Shanghai, China) and kept at a specific pathogen-free facility.

The nude mice were implanted subcutaneously with 1×10^6^ HepG2 cells. When the tumor diameters reach 5mm, the animals were intraperitoneally injected with doxorubicin (1 mg/kg, American Pharmaceutical Partners, USA). Doxorubicin was administered every 3 days for a total of eight dosages (continuous low-dose schedule dosage). 1×10^5^ MSC.sFlt1 were delivered intravenously to the mice 24hrs after MSC transduction with adv-sFlt1. The injections with 1×10^5^ MSC.sFlt1 were repeated two weeks later. Combined treatment consisted of at the beginning of 1×10^5^ MSC.sFlt1 treatment, 1 mg/kg doxorubicin given once every 3 days for a total of eight times.

The animals were randomly assigned into four groups (n=12/group): (i) saline group (ii) continuous low-dose doxorubicin group (iii) MSC.sFlt1group (iV) MSC.sFlt1 plus continuous low-dose doxorubicin group. Tumor diameters were measured with a caliper and tumor volumes were calculated by using the equation V(mm^3^)=0.5×a×b^2^, where a=length, b=width. All procedures with animals were reviewed and approved by the Instructional Animal Care and Use Committee at Xuzhou Medical University.

The mice were sacrificed 42 days after HepG2 cells injection. The tumors were excised. A section of the tumor was fixed in paraformaldehyde (4%) and paraffin-embedded, and HE was performed. Tissue specimens were also used for immunohistochemical analyses. The animal study was repeated independently once.

### Immunohistochemistry for expression of PCNA and microvessel density (MVD)

Tumor tissue sections (5 μm) were prepared for immunohistochemistry following reports [38]. Mouse anti-PCNA monoclonal antibody (1:100) (Leica Biosystems, UK) or goat anti-mouse CD31 polyclonal antibody (1:100) (Santa Cruz, USA) were used as primary antibodies. PBS instead of primary antibody was used as a negative control. The intensity of PCNA immunostaining was quantified using a computerized imaging system with 5 randomly selected fields at 200 times magnification. Staining was considered negative when the percentage of cells positive for PCNA staining was less than 10%. For MVD quantitation, 5 fields containing CD31 staining were selected and the number of CD31 positive cells in a field was quantified.

### Western blot

Western blots were principally carried out as described previously [35]. In brief, the protein lysate was mixed with loading buffer, boiled for 5 mins, and loaded into sodium dodecyl sulfate-polyacrylamide gels (SDS-PAGE) with equal amounts of protein. The gel was run at 200 V for 60mins, followed by transfer to nitro-cellulose membrane (Amersham Biosciences, USA) at 100 V for 30 min at room temperature and incubation with primary antibodies. The primary antibodies include anti-caspase8 (1:100), anti-wtP53 (1:100) (Santa Cruz Biotechnology, CA). The secondary antibodies, anti-goat/rabbit/mouse immunoglobulin G (IgG)-HRP, were purchased from Zhongshan Company (Beijing, China). The protein expression levels were detected by chemiluminescence (ECL system, Amersham, UK) and quantified using Quantity One software (Bio-Rad). The expression level of the genes of interest were normalized using β-actin.

### pSUPER-siRNA constructs and stable cell lines generation

The caspase8 siRNA and wtP53 siRNA hairpin oligos were designed by Ambion website (www.ambion.com) and synthesized by Shanghai SBS Genetech. Inc. (Shanghai, China). The sense strand oligo sequences were: caspase8: 5′-GGGTCATGCTCTATCAGAT-3′ and wtP53: 5′-TCTGTGACTTGCACGTACTTT-3′. The synthesized siRNA specific to the caspase8 or wtP53 gene was inserted into pSUPER vector according to our previous procedures[39], which was co-transfected with pSUPER-caspase8-siRNA or pSUPER-P53-siRNA and pEGFP vectors using Lipofectamine 2000 (Invitrogen, USA) to generate the HepG2- caspase8-siRNA or HepG2- P53-siRNA cell line. Clones that did not show a knock-down were used as controls.

### Statistical analysis

The results were expressed as means ± standard error of means of at least 3 independent experiments, unless stated otherwise. Experimental values are expressed as mean value ±SD. For significance analyses, analysis of variance (ANOVA) between groups was used (SAS 6.12, SAS Institute, USA).*P<0.05 was considered significant.

## Abbreviations

BCA: Bicinchoninic acid
FBS: Fetal bovine serum
PBS: Phosphate buffered saline
PVDF: Polyvinylidene difluoride
HE: Hematoxylin and eosin
MTT 3: 4,5- dimethylthiazol-2yl)-2,5-diphenyltetra-zoliumbromide
VEGF: Vascular endothelial growth factor
Flk-1/KDR: fetal liver kinase-1/kinase insert domain containing receptor
MSCs: Mesenchymal stem cells
HUVEC: human umbilical vein endothelial cells
Zvad: carbobenzoxy-valyl-alanyl-aspartyl-[O-methyl]-fluo-romethylketone
PCNA: nuclear proliferating cell nuclear antigen
MVD: microvessels density
CM: concentrated conditioned medium
sFlt1: soluble Fms-like tyrosine kinase-1
